# Assessment of Pelvic Floor Disorders due to the Gestational Diabetes Mellitus Using Three-Dimensional Ultrasonography: A Narrative Review

**DOI:** 10.1055/s-0042-1759742

**Published:** 2022-12-29

**Authors:** Carlos Izaias Sartorão Filho, Angélica Mércia Pascon Barbosa, Iracema de Mattos Paranhos Calderon, Marilza Vieira Cunha Rudge

**Affiliations:** 1 Department of Gynecology and Obstetrics, Faculdade de Medicina de Botucatu, Universidade Estadual de São Paulo (UNESP), Botucatu, SP, Brazil; 2 Department of Medical School, Fundação Educacional do Município de Assis (FEMA), Assis, SP, Brazil

**Keywords:** diabetes, gestational, pelvic floor, pelvic floor disorders, urinary incontinence, ultrasonography, diabetes gestacional, assoalho pélvico, desordens do assoalho pélvico, incontinência urinária, ultrassonografia

## Abstract

Gestational diabetes mellitus (GDM)is an entity with evolving conceptual nuances that deserve full consideration. Gestational diabetes leads to complications and adverse effects on the mother's and infants' health during and after pregnancy. Women also have a higher prevalence of urinary incontinence (UI) related to the hyperglycemic status during pregnancy. However, the exact pathophysiological mechanism is still uncertain. We conducted a narrative review discussing the impact of GDM on the women's pelvic floor and performed image assessment using three-dimensional ultrasonography to evaluate and predict future UI.

## Resumo

O diabetes gestacional (DG)é uma entidade com nuances conceituais em evolução que merecem total consideração. O DG leva a complicações e efeitos adversos na saúde da mãe e do bebê durante e após a gravidez. As mulheres também apresentam maior prevalência de incontinência urinária (IU) relacionada ao estado hiperglicêmico durante a gravidez. No entanto, o mecanismo fisiopatológico exato ainda é incerto. Realizamos uma revisão narrativa discutindo o impacto do DG no assoalho pélvico das mulheres e utilizamos o exame de ultrassonografia tridimensional para avaliar e predizer a ocorrência de IU.

## Introduction

We performed a narrative review of the literature with the intention of summarizing a qualitative interpretation of the prior knowledge towards gestational diabetes mellitus (GDM), the implication of the hyperglycemia status to the pelvic floor, and the outcome of postpartum urinary incontinence (UI). Moreover, we also evaluated the importance of the pelvic floor assessment using three-dimensional ultrasonography. In the last few decades, the Diamater Research Group, located at Faculdade de Medicina de Botucatu at Universidade Estadual de São Paulo, has been studying these important physiopathological mechanisms and assessment tools related to pelvic floor disorders. Their primary goal is to synthesize the extent of the body of knowledge regarding these particular research topics. We selected studies that support the critical findings in these areas. Hence, using the method of narrative review, we did not intend to formally assess the quality or the risk of bias in the literature provided.

## Gestational Diabetes Mellitus: An Entity with Evolving Conceptual Nuances

Gestational diabetes mellitus is defined as hyperglycemia first detected during pregnancy, with glycemic blood levels that do not meet the diagnostic criteria for diabetes mellitus (DM). 1 It differs from diabetes mellitus (DM) diagnosed during pregnancy, also called overt diabetes, which is when women, without a prior diagnosis, have hyperglycemia detected during pregnancy and present blood glycemic levels that meet the World Health Organization (WHO) criteria for DM in the absence of pregnancy. 1 Brazil has high rates of DM in the adult population, with an estimated total of 14.3 million people aged 20 to 79 years. The population estimated prevalence of hyperglycemia during pregnancy in Brazil is approximately 18%, using the diagnostic criteria currently proposed in the literature. 2

The International Association of Diabetes and Pregnancy Study Groups (IADPSG) defines that if the pregnant woman presents, in the first prenatal consultation, diagnostic criteria equal to those predetermined for the diagnosis of diabetes outside pregnancy (glycated hemoglobin ≥ 6.5%; fasting glycemia ≥ 126 mg/dL, or glycemia at any time ≥ 200 mg/dL), she will be considered as a carrier of previous DM or overt diabetes, diagnosed in pregnancy. 3 It also defines that the GDM diagnosis should be established when fasting glucose is ≥ 92 mg/dL and < 126 mg/dL. Alternatively, at least one of the values of the oral glucose tolerance test with 75 g (75g-OGTT), performed between 24 and 28 weeks of gestational age, is ≥ 92 mg/dL at fasting; ≥ 180 mg/dL in the 1st hour; and ≥ 153 mg/dL in the 2nd hour. The 75g-OGTT is universally recommended for all pregnant women who did not present previous DM or hyperglycemia at the beginning of pregnancy. 4 The HAPO study determined the cutoff points of the 75g-OGTT because they corresponded to an increase in the odds ratio of 1.75 for one of the following neonatal outcomes studied: birth weight above the 90th percentile, percentage of neonatal body fat above the 90th percentile, or C-peptide value in the umbilical cord above the 90th percentile. Thus, pregnant women with one or more points in the 75g-OGTT would have a 75% higher risk of having a newborn with one of these three neonatal outcomes when compared to pregnant women without any of these altered values. 5 6 Given the need to move towards a single criterion for the diagnosis of GDM, the WHO adopted the IADPSG. Two warnings were inserted: 1) that these criteria were valid for any gestational age, and 2) that the blood glucose value of 2 hours of 75g-OGTT should be between 153 and 199 mg/dL for the diagnosis of GDM since values ≥ 200 mg/dL correspond to the diagnosis of DM. 7 8

In prenatal routine, fasting glucose is recommended up to 20 weeks of gestational age to diagnose GDM and overt diabetes. All pregnant women with fasting glucose below 92 mg/dL should perform 75g-OGTT from 24 to 28 weeks. If the onset of prenatal care is delayed after 20 weeks of gestational age, 75g-OGTT should be completed as soon as possible. 1

Gestational diabetes mellitus leads to complications and adverse effects on the mother's and infant's health during pregnancy. In addition, in the immediate postpartum period, it can delay the onset of breastfeeding and affect the health of the woman and the infant. 9 Women diagnosed with GDM in the first half of pregnancy represent a high-risk subgroup for increased obstetric and clinical complications. 10 11 Women with GDM have a higher chance of recurrence of GDM in future pregnancies and also a higher risk of developing type 2 DM (T2DM) throughout life. Those with obesity or who require insulin for glycemic control during pregnancy have a higher risk of T2DM. Insulin resistance is the pathophysiological basis of both GDM and T2DM and can be addressed with measures that lead to increased insulin sensitivity, such as nutritional adequacy, exercise, and medications. These interventions reduce the risk of T2DM in high-risk women, such as those with a previous history of GDM. 12

## Gestational Diabetes and Postpartum Urinary Incontinence: A Neglected but Common Association

Urinary incontinence (UI) is defined by the International Continence Society as any involuntary loss of urine. 13 It is associated with patients' physical, psychological, and social discomforts. In addition, there are well-established risk factors for UI, including advanced age, obesity, and vaginal delivery. 14

A systematic review and meta-analysis conducted by Tähtinen et al., 14 in 2016, reported that vaginal delivery is associated with almost twice as long-term UI, an increase of about 8% when compared to cesarean delivery.

Gyhagen et al. 15 conducted a national cohort study in Sweden to investigate UI's prevalence and risk factors 20 years after a vaginal delivery or cesarean section. The study population consisted of 5,236 women who returned the questionnaire by mail, primiparous with a single pregnancy, had vaginal or cesarean delivery between 1985 and 1988, and had no later births. The prevalence of UI was higher after vaginal delivery (40.3%) than after cesarean section (28.8%); odds ratio (OR) 1.67; 95% confidence interval (CI) 1.45–1.92. In addition, there was an 8% increase in UI risk for each unit of body mass index (BMI) plus, and maternal age at delivery increased the risk of UI by 3% each year.

The weakening of the pelvic floor muscle (PFM) causes hypermobility of the bladder neck, and urethra, leading to the woman's incompetence of the urethral sphincter. Pregnancy itself is a significant risk factor for UI. The exact causes associated with pregnancy remain not fully understood. 16

During pregnancy, UI is more frequent as pregnancy progresses, compromising women's quality of life. There are few publications on the prevalence of the pregnancy-specific UI (PS-UI). In addition, little is known about the clinical implications regarding the time of onset of UI during pregnancy, and the factors involved in its pathophysiology remain unexplored. 17 A study conducted in Norway by Wesnes et al. 18 described a cumulative incidence of 46% of pregnant women with UI, and multiparity was the main and most relevant risk factor.

In a large cohort study with 81,845 women to assess the association between type 2 DM and UI, development risk was higher in diabetic women. 19 Women with GDM also have a higher prevalence of UI. However, the exact pathophysiological mechanism is still uncertain. Nevertheless, weight gain, obesity, fetal macrosomia, and any conditions that increase bladder pressure and urethral mobility may be implicated. In addition, hyperglycemia can cause polyuria and detrusor instability. Hence, the risk of UI is higher during pregnancy and persists after childbirth. 20 Kim et al. 20 examined the prevalence of UI among women with GDM. They found that 49% of women reported urine loss during pregnancy, and 50% reported UI in the first 5 years after delivery. 20 Chuang et al., 21 in a survey of 6,653 women with GDM, described that incontinent women who had GDM had a higher severity of UI 2 years after delivery. Thus, they conclude that GDM is an independent risk factor for postpartum UI, with an essential impact on the severity of symptoms. 21

A pioneer cross-sectional study in Brazil conducted by Barbosa et al. 22 with 832 selected women evaluated the prevalence of 2-years postpartum UI and found it was 18.9% after cesarean section and 17% after vaginal delivery, with no statistical differences between delivery routes. Women who had increased weight gain during pregnancy were at increased risk for PF dysfunction during pregnancy. Women with GDM had a significantly higher UI prevalence 2-years postpartum (OR: 8.6, 95% CI: 3.0–24.3). 22

## The Pelvic Floor

The PF deep muscles consist of the levator ani muscle (LAM), formed by the puborectalis, pubococcygeus, and iliococcygeus muscles. The superficial muscles of the PF form the urogenital diaphragm and include the cavernous ischium, spongy bulb, and the superficial transverse muscle of the perineum. Fascia interposes these muscles continued with the pelvic endofascia, which involves the pelvic viscera, and contributes to the PF support. 23

The LAM has a tapered shape, with a central slit through the urethra, vagina, and anus. The puborectalis part is the lowest and is placed in the lower branches of the pubis and later borders the anal canal. The function of the PF muscles is to make voluntary and involuntary contractions, responsible for urinary and fecal continence. The puborectalis portion of LAM is essential in supporting and conserving continence. 24

Pelvic floor disorders (PFDs), including UI, genital prolapse, and anal incontinence, are highly prevalent in women of all ages. Imaging evaluation methods are essential for diagnosing and treating diseases and studying the integrity of pelvic structures. Many imaging modalities are used to evaluate the PF, such as computed tomography (CT), magnetic resonance imaging (MRI), and contrast-enhanced defecography. 25 As limitations, CT and defecography use contrasts and employ ionizing radiation. Defecography can replicate and evaluate patients' symptoms in real-time during defecation. However, it employs X-ray, it is unpleasant for the patient, and it is challenging to reproduce. Magnetic resonance imaging provides a good evaluation of the soft tissues of the PF without the use of ionizing radiation. However, it requires the use of contrasts, and it is not operator-dependent. In addition, the high cost of the examination, the prolonged time of image acquisition, and the difficulty of availability of the device limit its use in practice and are disadvantages. More importantly, MRI does not allow the proper evaluation of the functional maneuvers of the PF. 26 Ultrasound (US) imaging is widely used for morphological and functional evaluation of the PF. Studies have demonstrated the superiority of the US in conjunction with clinical evaluation compared with manometry, electromyography, and defecography. It is also helpful as a biofeedback tool for functional PF training. Anal ultrasound of the PF is beneficial for evaluating the anal sphincter and diagnosing fecal incontinence. It uses specific high-frequency transducers that ideally reproduce a 360-degree panoramic image to visualize the anal sphincter complex properly. Transvaginal US for PF evaluation employs identical transducers to study internal pelvic structures. However, it has limited use due to the very close proximity to the PF structures, the possible interference of the transducer in the functional evaluation, and especially the limited angle of insonation provided by the transducer for good acquisition of the images. 27

The transperineal, or translabial, US was one of the first ultrasound modalities used to study the PF. It is a handy and widely available tool, of low cost, little invasive, and easy to reproduce. It also allows the evaluation of the structures of the PF during functional maneuvers. The three-dimensional US (US3D) of the PF is a technique described more than 20 years ago which has gained more notoriety in recent years. It reproduces three-dimensional images of the PF similarly to those obtained by MRI, with the advantages of being more widely available and more affordable, being faster and mainly allowing clinical and functional evaluation of the patient in real-time. In addition, it does not cause more significant discomfort for the patient and does not require contrasts for its execution. It is less user-dependent than the two-dimensional US, which contributes to greater accuracy of the evaluations and measurements of the PF. The 3DUS provides an adequate and reliable assessment of the anatomy and function of muscles and structures, essential in the clinical and complementary diagnosis, treatment, and follow-up of PF disorders. Therefore, many authors argue that the PF 3DUS should be routinely used to evaluate and manage diseases and dysfunctions. In addition to assessing the structures and functions of LAM, imaging methods are essential to exclude coexisting diseases, propose individualized treatments according to the findings, monitor therapy, better understand therapeutic failures, and support the study of conditions that can cause damage to the LAM. 28

The comparison between MRI and 3DUS is frequently found in the literature, and the benefits and practicality of using the PF 3DUS are well established and validated. 29

The etiology of PF disorders is multifactorial. Traumatic damage to support structures during labor and vaginal delivery may be important factors contributing to UI and genital prolapse development. 30

In 2009, Shek and Dietz, 31 using 3DUS from the PF before and after delivery, concluded that vaginal delivery results in enlargement of the hiatal area (HA), especially after LAM avulsion. However, even without macroscopic alteration of the muscle, there may be greater distensibility of the HA, which may be related to other mechanisms. 31

The same authors, in 2010, conducted a prospective longitudinal study in 468 nulliparas in the 3rd trimester and after delivery to determine whether the prediction of trauma to the LAM is feasible with the 3DUS, without success. They concluded that prediction is very difficult or even impossible. 32

In a prospective longitudinal study in Germany, in 2013, Falkert et al. 33 used 3DUS after immediate delivery and 18 to 24 months postpartum. The objective was to evaluate whether the changes observed in the PF after prompt delivery persisted after 18 to 24 months. A total of 59% of women completed the follow-up, and a significant increase in HA was observed in vaginal postpartum compared to cesarean section. However, there were no significant UI changes between the vaginal and cesarean groups. Independently of the mode of delivery, the UI incidence was higher in the larger HA group. 33

A study conducted in Brazil, in 2013, by Araujo Júnior et al. 34 at Universidade Federal de São Paulo evaluated the changes in the 3DUS of the PF of primiparous women with different delivery modes. They demonstrated higher HA in the postpartum vaginal group and forceps about cesarean delivery. 34

Chan et al., in 2013, 35 investigated PF biometrics during pregnancy and its correlation with symptoms of PF disorders in each trimester of pregnancy. The HA significantly increased by 15.1 +/- 24.8% at rest and 24.7 +/- 28.5% at Valsalva from the first to the third trimester. Symptoms of UI, bladder neck descent, and prolapse were associated with increased HA. 35

Siafarikas et al. 36 investigated the association between the PF dimensions at the end of the pregnancy with the second stage of labor duration and the type of delivery. In conclusion, they found a significant association between HA and the shorter duration of the active phase of the second delivery stage and expected vaginal delivery. However, the process of parturition is highly complex, and the pelvic anatomy is only an influencing factor. The clinical findings are inconclusive in determining the risk predictors for dystocic or instrumentalized deliveries. 36 In contrast, Van Veelen et al. 37 showed that smaller HA dimensions during the contraction of the LAM in the first pregnancy were associated with instrumentalized or cesarean delivery. In another study, Van Veelen et al. 38 demonstrated that the HA values and the contractility and distensibility of the LAM increase during the first pregnancy. Thus, regardless of the type of delivery, this more significant distension of the HA persists after birth and may be related to future PF dysfunctions in the woman's life. 38

Staer-Jensen et al. 39 studied the morphological changes of the PF in a cohort of primiparous women. In conclusion, the LAM can recover after pregnancy and delivery, although not all women recover from the levels demonstrated during pregnancy. 39

Siafarikas et al. (2013) 40 published a study on the learning process to perform and analyze the images of the PF 3DUS. They concluded that the exam can be learned quickly and that the technique is reliable. 40 The publication addresses the length of the learning process for multiple measures of hiatal functional anatomy, showing that the measurement of all assessed hiatal dimensions could be taught to an acceptable standard within 23 hours of total training, confirming several other studies demonstrating good repeatability of levator hiatal dimensions.

We published a study by Sartorão Filho et al. 41 that evaluated the PF biometry using 3DUS at 2 time points of gestation in pregnant women with GDM. We performed a prospective cohort study at the Perinatal Diabetes Research Center, including 44 pregnant women with GDM and 66 pregnant women without GDM at 24 to 28 weeks of gestation. The minimal hiatal dimensions plane was used to determine the HA biometry at 24 to 28 and 34 to 38 weeks of pregnancy by 3DUS. Of a 110 pregnant women, 100 (90.9%) completed the follow-up. The 3DUS measurements showed a negative biometric change between the 2 time points in pregnancy in women with GDM; in the HA (β coefficient: estimative of effect in biometric progression according to GDM diagnosis, using the non-GDM group as reference = − 6.76; *P* = .020), anteroposterior diameter (β = − 5.07; *P* = .019), and levator ani thickness (β = − 12.34; *P* = 0.005). Pregnant women with GDM had a significantly lower than expected percentage of changes in biometry of levator ani thickness and HA from 24 to 28 to 34 to 38 weeks of gestation when compared with the group of pregnant women with non-GDM. Thus, GDM altered the biometric morphology of PF structures assessed by the 3DUS. This reported complication may be implicated in adverse birth outcomes and may play a role in developing PF dysfunction. 41

## The Pelvic Floor 3DUS Exam Technique

**Fig. 1:**
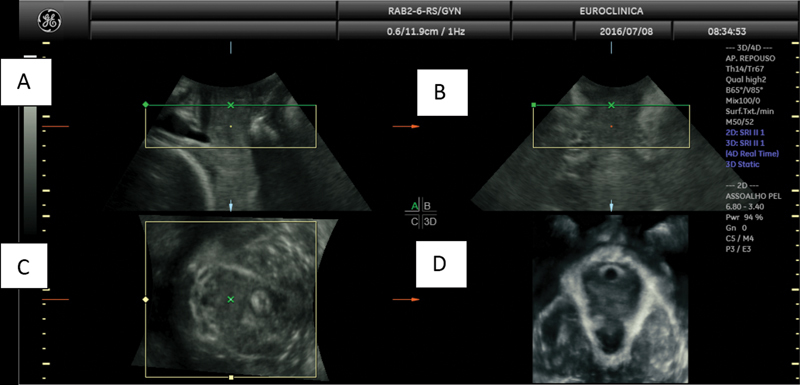
Image of the three orthogonal planes: A - Mid-sagittal plane, B - Coronal plane, C - Axial plane, D - Axial plane with the rendered image.

The US3D biometry data of the PF used by the Diamater study group were anteroposterior diameter, transversal diameter, and HA, collected at rest, during maximum contraction, and maximum Valsalva maneuver. Women were positioned in the lithotomy position after voiding. The equipment used was the GE P8 or the GE Voluson i system with a 2-to-6 MHz curved array three-dimensional transducer (GE Healthcare, Zipf, Austria). We acquired the volume angle setting maximum in the sagittal plane and 85° in the coronal plane. Offline analysis of the rendered volume datasets was blinded using the 4D View (GE Healthcare) software program. Finally, we used the method proposed by Dietz, 27 obtaining the image of the three orthogonal planes as seen in Figure 1 .

**Fig. 2:**
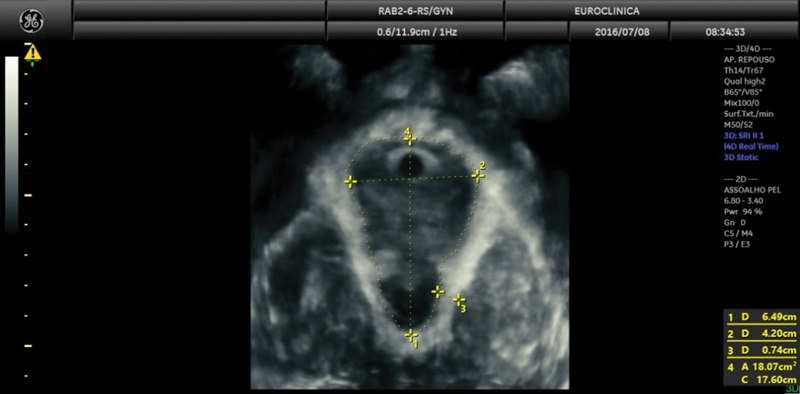
Axial plane of pelvic floor: 1 - anteroposterior diameter, 2 - transverse diameter, and 3 - Levator ani muscle thickness 4 - hiatal area.

Figure 2 demonstrates the levator hiatal dimensions, measured in the axial plane of minimal levator hiatal distances, identified in the mid-sagittal image as the minimal distance between the inferior margin of the symphysis pubis and the anorectal junction. The anteroposterior diameter of the levator hiatus was defined as the minimum distance in a mid-sagittal direction and was measured from the symphysis pubis' inferior border to the levator's posterior margin ani. The levator hiatal transverse diameter was measured at its widest part from the internal border of the levator ani muscle, perpendicular to the anteroposterior diameter. The levator hiatus area was measured as the internal area bordered by the LAM, pubic symphysis, and the inferior pubic ramus. 27 The LAM's thickness is another possible measurement, as shown in Figure 1 , although we did not perform or consider it for our research. The transperineal 3DUS learning process is reliable, repeatable, and practical. Thus, it should be incorporated into the modern arsenal of PF evaluation.

## References

[1] Organização Pan-Americana da Saúde Ministério da Saúde. Federação Brasileira das Associações de Ginecologia e ObstetríciaSociedade Brasileira de Diabetes. Rastreamento e diagnóstico de diabetes mellitus gestacional no Brasil [Internet]. Brasília (DF): OPAS; 2017 [cited 2021 Jul 22]. Available from:https://iris.paho.org/bitstream/handle/10665.2/34278/9788579671180-por.pdf?sequence=1&isAllowed=y

[2] Trujillo J Vigo A Duncan B B Falavigna M Wendland E M Campos M A Schmidt M I Impact of the International Association of Diabetes and Pregnancy Study Groups criteria for gestational diabetes Diabetes Res Clin Pract 2015 108 02 288 295 10.1016/j.diabres.2015.02.007 25765668

[3] McIntyre H D Colagiuri S Roglic G Hod M Diagnosis of GDM: a suggested consensus Best Pract Res Clin Obstet Gynaecol 2015 29 02 194 205 10.1016/j.bpobgyn.2014.04.022 25242583

[4] Laafira A White S W Griffin C J Graham D Impact of the new IADPSG gestational diabetes diagnostic criteria on pregnancy outcomes in Western Australia Aust N Z J Obstet Gynaecol 2016 56 01 36 41 10.1111/ajo.12394 26293845

[5] International Association of Diabetes & Pregnancy Study Groups (IADPSG) Consensus Panel Writing Group and the Hyperglycemia & Adverse Pregnancy Outcome (HAPO) Study Steering Committee. The diagnosis of gestational diabetes mellitus: new paradigms or status quo? J Matern Neonatal Med. 2012 25 12 2564 2569 10.3109/14767058.2012.7180022 22876884

[6] HAPO Study Cooperative Research Group Lowe L P Metzger B E Dyer A R Lowe J McCance D R Lappin T RJ Hyperglycemia and Adverse Pregnancy Outcome (HAPO) Study: associations of maternal A1C and glucose with pregnancy outcomes Diabetes Care 2012 35 03 574 580 10.2337/dc11-1687 22301123 PMC3322718

[7] Hod M Kapur A Sacks D A Hadar E Agarwal M Di Renzo G C FIGO initiative on gestational diabetes mellitus: a pragmatic guide for diagnosis, management, and care Int J Gynaecol Obstet 2015 131 03 S173 S211 10.1016/S0020-7292(15)30033-3 29644654

[8] Kapur A Mahmood T Hod M FIGO's response to the global challenge of hyperglycemia in pregnancy - toward a global consensus Gynecol Endocrinol 2018 34 01 1 3 10.1080/09513590.2017.1381682 28980832

[9] Goedegebure E AR Koning S H Hoogenberg K Korteweg F J Lutgers H L Diekman M JM Pregnancy outcomes in women with gestational diabetes mellitus diagnosed according to the WHO-2013 and WHO-1999 diagnostic criteria: a multicentre retrospective cohort study BMC Pregnancy Childbirth 2018 18 01 152 10.1186/s12884-018-1810-5 29747601 PMC5946499

[10] Riskin-Mashiah S Younes G Damti A Auslender R First-trimester fasting hyperglycemia and adverse pregnancy outcomes Diabetes Care 2009 32 09 1639 1643 10.2337/dc09-0688 19549728 PMC2732138

[11] Gupta S Dolin C Jadhav A Chervenak J Timor-Tritsch I Monteagudo A Obstetrical outcomes in patients with early onset gestational diabetes J Matern Fetal Neonatal Med 2016 29 01 27 31 10.3109/14767058.2014.991711 25424373

[12] Dickens L T Thomas C C Updates in gestational diabetes prevalence, treatment, and health policy Curr Diab Rep 2019 19 06 33 10.1007/s11892-019-1147-0 31073850

[13] International Urogynecological Association International Continence Society Haylen B T de Ridder D Freeman R M Swift S E Berghmans B Lee J An International Urogynecological Association (IUGA)/International Continence Society (ICS) joint report on the terminology for female pelvic floor dysfunction Neurourol Urodyn 2010 29 01 4 20 10.1007/s00192-009-0976-9 19941278

[14] Tähtinen R M Cartwright R Tsui J F Aaltonen R L Aoki Y Cárdenas J L Long-term impact of mode of delivery on stress urinary incontinence and urgency urinary incontinence: a systematic review and meta-analysis Eur Urol 2016 70 01 148 158 10.1016/j.eururo.2016.01.037 26874810 PMC5009182

[15] Gyhagen M Bullarbo M Nielsen T F Milsom I The prevalence of urinary incontinence 20 years after childbirth: a national cohort study in singleton primiparae after vaginal or caesarean delivery BJOG 2013 120 02 144 151 10.1111/j.1471-0528.2012.03301.x 22413831

[16] Sangsawang B Sangsawang N Stress urinary incontinence in pregnant women: a review of prevalence, pathophysiology, and treatment Int Urogynecol J Pelvic Floor Dysfunct 2013 24 06 901 912 10.1007/s00192-013-2061-7 PMC3671107 23436035

[17] Martínez Franco E Parés D Lorente Colomé N Méndez Paredes J R Amat Tardiu L Urinary incontinence during pregnancy. Is there a difference between first and third trimester? Eur J Obstet Gynecol Reprod Biol 2014 182 86 90 10.1016/j.ejogrb.2014.08.035 25262291

[18] Wesnes S L Rortveit G Bø K Hunskaar S Urinary incontinence during pregnancy Obstet Gynecol 2007 109 04 922 928 10.1097/01.AOG.0000257120.23260.00 17400855

[19] Lifford K L Curhan G C Hu F B Barbieri R L Grodstein F Type 2 diabetes mellitus and risk of developing urinary incontinence J Am Geriatr Soc 2005 53 11 1851 1857 10.1111/j.1532-5415.2005.53565.x 16274364

[20] Kim C McEwen L N Sarma A V Piette J D Herman W H Stress urinary incontinence in women with a history of gestational diabetes mellitus J Womens Health (Larchmt) 2008 17 05 783 792 10.1089/jwh.2007.0616 18537481 PMC2942747

[21] Chuang C M Lin I F Horng H C Hsiao Y H Shyu I L Chou P The impact of gestational diabetes mellitus on postpartum urinary incontinence: a longitudinal cohort study on singleton pregnancies BJOG 2012 119 11 1334 1343 10.1111/j.1471-0528.2012.03468.x 22901044

[22] Barbosa A M Dias A Marini G Calderon I M Witkin S Rudge M V Urinary incontinence and vaginal squeeze pressure two years post-cesarean delivery in primiparous women with previous gestational diabetes mellitus Clinics (São Paulo) 2011 66 08 1341 1346 10.1590/s1807-59322011000800006 21915481 PMC3161209

[23] Dietz H P Shek C Clarke B Biometry of the pubovisceral muscle and levator hiatus by three-dimensional pelvic floor ultrasound Ultrasound Obstet Gynecol 2005 25 06 580 585 10.1002/uog.1899 15883982

[24] Durnea C M O'Reilly B A Khashan A S Kenny L C Durnea U A Smyth M M Dietz H P Status of the pelvic floor in young primiparous women Ultrasound Obstet Gynecol 2015 46 03 356 362 10.1002/uog.14711 25359670

[25] Chamié L P Ribeiro D MFR Caiado A HM Warmbrand G Serafini P C Translabial US and dynamic MR imaging of the pelvic floor: normal anatomy and dysfunction Radiographics 2018 38 01 287 308 10.1148/rg.2018170055 29320316

[26] Dietz H P Pelvic floor assessment Fetal Matern Med Rev 2009 20 01 49 66 10.1017/S096553950900237X

[27] Dietz H P Pelvic floor ultrasound: a review Clin Obstet Gynecol 2017 60 01 58 81 10.1097/GRF.0000000000000264 28005595

[28] Dietz H P Pelvic floor ultrasound: a review Am J Obstet Gynecol 2010 202 04 321 334 10.1016/j.ajog.2009.08.018 20350640

[29] Majida M Braekken I H Bø K Benth J S Engh M E Validation of three-dimensional perineal ultrasound and magnetic resonance imaging measurements of the pubovisceral muscle at rest Ultrasound Obstet Gynecol 2010 35 06 715 722 10.1002/uog.7587 20178105

[30] Memon H U Handa V L Vaginal childbirth and pelvic floor disorders Womens Health (Lond Engl) 2013 9 03 265 277, quiz 276–277. Doi: 10.2217/whe.13.17 23638782 10.2217/whe.13.17 PMC3877300

[31] Shek K L Dietz H P The effect of childbirth on hiatal dimensions Obstet Gynecol 2009 113 06 1272 1278 10.1097/AOG.0b013e3181a5ef23 19461422

[32] Shek K L Dietz H P Can levator avulsion be predicted antenatally? Am J Obstet Gynecol 2010 202 06 5860 5.86E8 10.1016/j.ajog.2009.11.038 20079479

[33] Falkert A Willmann A Endress E Meint P Seelbach-Göbel B Three-dimensional ultrasound of pelvic floor: is there a correlation with delivery mode and persisting pelvic floor disorders 18-24 months after first delivery? Ultrasound Obstet Gynecol 2013 41 02 204 209 10.1002/uog.11214 22745047

[34] Araujo Júnior E de Freitas R C Di Bella Z I Alexandre S M Nakamura M U Nardozz L MM Moron A F Assessment of pelvic floor by three-dimensional-ultrasound in primiparous women according to delivery mode: initial experience from a single reference service in Brazil Rev Bras Ginecol Obstet 2013 35 03 117 122 10.1590/s0100-72032013000300005 23538470

[35] Chan S S Cheung R Y Yiu K W Lee L L Leung T Y Chung T K Pelvic floor biometry during a first singleton pregnancy and the relationship with symptoms of pelvic floor disorders: a prospective observational study BJOG 2014 121 01 121 129 10.1111/1471-0528.12400 24148651

[36] Siafarikas F Staer-Jensen J Hilde G Bø K Ellström Engh M The levator ani muscle during pregnancy and major levator ani muscle defects diagnosed postpartum: a three- and four-dimensional transperineal ultrasound study BJOG 2015 122 08 1083 1091 10.1111/1471-0528.13332 25716540

[37] van Veelen G A Schweitzer K J van Hoogenhuijze N E van der Vaart C H Association between levator hiatal dimensions on ultrasound during first pregnancy and mode of delivery Ultrasound Obstet Gynecol 2015 45 03 333 338 10.1002/uog.14649 25158301

[38] van Veelen G A Schweitzer K J van der Vaart C H Ultrasound imaging of the pelvic floor: changes in anatomy during and after first pregnancy Ultrasound Obstet Gynecol 2014 44 04 476 480 10.1002/uog.13301 24436146

[39] Stær-Jensen J Siafarikas F Hilde G Benth JŠ Bø K Engh M E Postpartum recovery of levator hiatus and bladder neck mobility in relation to pregnancy Obstet Gynecol 2015 125 03 531 539 10.1097/AOG.0000000000000645 25730212

[40] Siafarikas F Staer-Jensen J Braekken I H Bø K Engh M E Learning process for performing and analyzing 3D/4D transperineal ultrasound imaging and interobserver reliability study Ultrasound Obstet Gynecol 2013 41 03 312 317 10.1002/uog.11192 22605574

[41] Sartorão Filho C I Pinheiro F A Prudencio C B Nunes S K Takano L Enriquez E MA Impact of gestational diabetes on pelvic floor: A prospective cohort study with three-dimensional ultrasound during two-time points in pregnancy Neurourol Urodyn 2020 39 08 2329 2337 10.1002/nau.24491 32857893

